# Protective Effects of *Magnolia kobus* DC. Extract on Inflammatory Response and Alveolar Bone Loss in Ligature-Induced Periodontitis Rats

**DOI:** 10.3390/cimb48010109

**Published:** 2026-01-20

**Authors:** Da-Eun Min, Sung-Kwon Lee, Eunji Kim, Seong-Hyeon Park, Deok-Geun Kim, Bong-Keun Choi

**Affiliations:** Research Institute, NUON Co., Ltd., Seongnam 13201, Republic of Korea; demin@nuon.kr (D.-E.M.); sklee@nuon.kr (S.-K.L.); dms3003@nuon.kr (E.K.); shpark@nuon.kr (S.-H.P.); ejrrms0224@nuon.kr (D.-G.K.)

**Keywords:** Magnolia, periodontitis, inflammation, alveolar bone loss, matrix metalloproteinases

## Abstract

Periodontitis is a chronic inflammatory condition characterized by dysregulated immune responses that promote alveolar bone destruction. Targeting inflammatory signaling pathways has therefore become an important area of investigation. This study investigated the anti-inflammatory and bone-protective effects of *Magnolia kobus* DC. extract (MKE) in a ligature-induced periodontitis rat model. Rats were assigned to five groups (*n* = 5 per group): non-ligature control, ligature control, doxycycline (20 mg/kg), MKE 100 mg/kg, and MKE 400 mg/kg, and treated orally for eight weeks. Periodontal damage and alveolar bone loss were assessed by micro-computed tomography (micro-CT), gingival index, and tooth mobility. Micro-CT analysis demonstrated a dose-dependent reduction in alveolar bone loss, as evidenced by a significant decrease in the cementoenamel junction–alveolar bone crest (CEJ–ABC) distance and reduced furcation involvement in MKE-treated groups compared with the ligature control group, while tooth mobility scores were significantly improved. Serum levels of receptor activator of nuclear factor kappa-B ligand, interleukin-1β, tumor necrosis factor-α, and cyclooxygenase-2 were significantly decreased, while nuclear factor kappa-B signaling was suppressed in gingival tissue. The extract also significantly reduced matrix metalloproteinases 3, 8, 9, and 13, and increased collagen type I and II expression. In summary, MKE exerted anti-inflammatory and bone-protective properties, effectively reducing alveolar bone loss and maintaining periodontal structure. These findings support MKE’s potential application as a natural anti-inflammatory and bone-protective agent and as a functional food ingredient for periodontitis prevention and treatment, meriting further clinical evaluation.

## 1. Introduction

Periodontitis is a chronic inflammatory disease that develops because of complex and multifactorial interactions between the subgingival biofilm and the host immune response [1]. Dysbiosis within the oral microbiota triggers innate and adaptive immune activation, leading to the production of pro-inflammatory cytokines and sustained inflammatory signaling in periodontal tissues [2]. Oral microbial dysbiosis arises from the combined effects of persistent plaque accumulation, periodontal pocket-associated microenvironmental changes, dysregulated host immune responses, and systemic risk factors, leading to the preferential expansion of pathogenic bacteria and sustained periodontal inflammation [3].

Once the inflammatory response is activated, macrophages and gingival fibroblasts secrete inflammatory cytokines such as interleukin (IL)-1β, IL-6, and tumor necrosis factor-α (TNF-α), thereby intensifying the immune response and stimulating the nuclear factor κ-light-chain-enhancer of activated B cells (NF-κB) as well as mitogen-activated protein kinase (MAPK) pathways [4]. The persistent inflammatory process contributes to alveolar bone resorption, which is a critical driver in the progression of periodontal tissue degradation by facilitating receptor activator of nuclear factor κ-Β ligand (RANKL)-dependent osteoclast activation [5]. As periodontitis advances, a continuous increase in osteoclastogenesis and heightened matrix metalloproteinase (MMPs) activity results in the progressive degradation of periodontal tissues and alveolar bone, which can ultimately culminate in tooth loss [6]. Consequently, the primary pathological targets for periodontitis are the suppression of inflammation and the prevention of alveolar bone loss [7]. Although mechanical debridement, including scaling, remains the first-line treatment for periodontitis, adjunctive pharmacological therapies such as antibiotics and anti-inflammatory agents are sometimes employed in specific clinical contexts; however, their prolonged use may increase the risk of antibiotic resistance and adverse effects, underscoring the need for safer and more effective natural product-based alternatives [8].

Recently, the increasing interest in natural product-based interventions has focused attention on the bioactive constituents of Magnolia species. Magnolia species are known to contain a diverse range of bioactive compounds such as magnolin, epimagnolin, fargesin, and aschantin, lirirresinol B dimethyl ether that exhibit anti-inflammatory, antimicrobial, and antioxidant activities, with prior studies documenting extensive pharmacological effects, including immune modulation, antioxidative, and antimicrobial properties [9,10,11,12]. Among these constituents, magnolin, a lignan compound found in *Magnolia* species, has been shown to possess potent biological activities, including anticancer, anti-inflammatory, and antioxidant effects [13,14,15]. Moreover, magnolin has been reported to inhibit the activation of the NF-κB signaling pathway in chondrocytes, thereby exerting strong anti-inflammatory effects that contribute to the alleviation of osteoarthritis [16].

Our previous in vitro study involving RAW 264.7 macrophages confirmed that *Magnolia kobus* DC. extract (MKE) effectively suppresses *Porphyromonas gingivalis* LPS-induced inflammatory responses [16]. In RAW 264.7 macrophages, MKE markedly decreased the expression levels of TNF-α, IL-1β, and IL-6. Moreover, it inhibited the NF-κB signaling pathway, contributing to modulation of the inflammatory response and suppressing RANKL-dependent osteoclastogenesis, indicating a possible role in mitigating bone resorption. Although previous research has shown that MKE inhibits in vitro inflammatory responses, evidence regarding its efficacy in improving periodontitis in animal models is currently inadequate [17].

Therefore, the aim of this study was to investigate the effects of orally administered MKE on periodontal inflammation and alveolar bone loss using a ligature-induced periodontitis rat model. Specifically, we evaluated whether systemic administration of MKE attenuates inflammatory cytokine expression and protects periodontal tissues from structural damage and alveolar bone resorption. In addition, the in vivo findings were interpreted in conjunction with previous in vitro studies to provide mechanistic insights into the anti-inflammatory activity of MKE for periodontitis.

## 2. Materials and Methods

### 2.1. Preparation of MKE

The MKE (Lot No. CWCH-A001756) utilized in this investigation was provided by NUON Co., Ltd. (Seongnam, Republic of Korea). The dried botanical material was subjected to extraction with water, then filtered, concentrated, and dried to obtain the final extract product. Quantitative HPLC analysis was carried out to assess the marker compound composition, with magnolin identified and quantified as the reference marker. The concentration of magnolin in MKE was determined to be approximately 1.5% of extract.

### 2.2. Experimental Animals

Male Sprague-Dawley rats, aged six weeks, were purchased from Orient Bio Inc. (Seongnam, Republic of Korea). Animals were housed in polycarbonate cages (W 170 × L 235 × H 125 mm) at a temperature of 23 ± 3 °C, relative humidity of 55 ± 15%, and a 12-h light–dark cycle, with illumination ranging from 150–300 Lux. A pelleted diet (Cargill Agri Purina via Dream Bio, Seoul, Republic of Korea), purified water (polycarbonate bottles), and wood shavings bedding (Coatech, Pyeongtaek) were provided ad libitum. All experimental procedures complied with national guidelines regarding laboratory animal care and were reviewed and approved by the Animal Ethics Committee of KNOTUS Co., Ltd. (Guri, Republic of Korea) under protocol KNOTUS-IACUC-17-KE-333. The rats underwent a 7-day acclimatization period before experimentation, during which they were monitored daily for abnormal health signs. Animals were randomly assigned to experimental groups after baseline body weights were measured. Animals were ordered by body weight and distributed across groups using a random allocation procedure that minimized differences in mean body weight at baseline. No predefined inclusion or exclusion criteria were applied after randomization, and all data points were included in the analysis. Cages rotated weekly. All animals (total 25 rats) completed the study and were included in the analyses.

### 2.3. Induction of Periodontitis and MKE Administration

To induce periodontitis, rats were anesthetized via intraperitoneal injection with Zoletil 50 (Virbac, Carros, France) and xylazine (Rompun^®^, Leverkusen, Germany). A 4-0 silk ligature was placed around the right second mandibular molar and retained for 8 weeks to establish the periodontitis model. Following induction, rats were randomly distributed into five groups (*n* = 5 per group): (1) non-ligature control + vehicle, (2) ligature control + vehicle, (3) ligature + doxycycline 20 mg/kg, (4) ligature + MKE 100 mg/kg, and (5) ligature + MKE 400 mg/kg. The non-ligature control, ligature control, and doxycycline-treated groups were shared reference groups within a single experimental cohort [18], while different Magnolia-derived extracts were evaluated in parallel as independent treatment arms.

The sample size was determined based on previous ligature-induced periodontitis studies demonstrating significant differences in alveolar bone loss and inflammatory markers with similar group sizes [19,20]. Although a formal power analysis was not conducted, the number of animals was considered sufficient to detect biologically relevant effects while adhering to ethical guidelines for animal research. MKE was dissolved in distilled water and administered by oral gavage once daily for 8 weeks. The gavage volume was calculated based on the most recently measured body weight of each rat at a dose of 10 mL/kg/day. Accordingly, the actual administered volume was individually adjusted for each animal and remained within the recommended safety limits for oral gavage. MKE was well-tolerated at the administered doses, with no observable adverse effects or changes in body weight or behavior throughout the study.

### 2.4. Micro-CT Analysis

To evaluate alveolar bone loss and associated tissue injury, all rats were anesthetized, and the mandibular jaws were scanned with a micro-CT system (SCANCO Medical, Brüttisellen, Switzerland) at 8 and 16 weeks after ligature placement to assess both established and long-term changes in alveolar bone morphology during periodontitis progression. The distance from the cementoenamel junction (CEJ) to the alveolar bone crest (ABC), along with the extent of furcation involvement in the regions affected by periodontitis, was determined from the scans. These quantitative assessments were conducted using the software integrated into the instrument. The mean CEJ–ABC distance was obtained by averaging the individual measurements between the CEJ and ABC at the left and right mandibular second molars. Furthermore, furcation involvement was evaluated using cross-sectional micro-CT images of the second molar region generated through this software. Scanning parameters were standardized to 70 kV with an energy output of 114 A and an integration time of 200 ms per projection. Approximately 420 consecutive image slices, each 25 μm thick, were acquired to provide continuous serial imaging from the incisor teeth to the mandible. These datasets were reconstructed with a voxel size of 25 μm, allowing for high-resolution structural analysis.

### 2.5. Assessment of Gingival Index and Tooth Mobility

The status of the gingiva and tooth mobility was monitored weekly after periodontitis induction by examining ligature placement, gingival bleeding, and the extent of soft tissue erosion. The degree of gingival inflammation was rated using the following criteria: Score 0—healthy gingiva without inflammatory signs; Score 1—mild inflammation indicated by slight swelling, minor changes in color, and absence of bleeding on probing; Score 2—moderate inflammation marked by swelling, glossiness, redness, and bleeding upon probing; Score 3—severe inflammation manifesting as marked redness, ulceration, swelling, and heavy bleeding. Severity of gingival inflammation was quantified using the Gingival Index. Tooth mobility evaluation followed a defined scoring system: Score 0—no detectable looseness; Score 1—slight mobility restricted to the vestibular–palatal direction; Score 2—moderate mobility observed in both vestibular–palatal and mesial–distal planes; Score 3—severe mobility characterized by vertical displacement, with the tooth demonstrating movement into and out of its socket. All evaluations were conducted under blinded conditions to minimize observer bias and ensure the reliability of the results.

### 2.6. Serum Analysis

Upon completion of the 16-week experimental period, rats were anesthetized, and blood was collected. The samples were subsequently centrifuged at 2000× *g* for 15 min at 4 °C to obtain the serum fraction. Serum samples were stored at −80 °C prior to biochemical assessment. Levels of IL-1β, TNF-α (Invitrogen, Carlsbad, CA, USA), cyclooxygenase-2 (COX-2; CUSABIO, Houston, TX, USA), and RANKL (LS Bio, Seattle, WA, USA) in the serum were quantified using commercial assay kits, in accordance with the manufacturer’s instructions.

### 2.7. Analysis of the Expression Levels of Periodontitis-Related Factors in Periodontal Tissue

After the 16-week study period, the rats were euthanized, and the adjacent tooth tissues were rapidly harvested and stored at −80 °C. Protein expression was evaluated employing Western blotting methods. The collected tissues were homogenized in lysis buffer supplemented with protease and phosphatase inhibitors, and subsequently centrifuged at 12,000× *g* for 20 min at 4 °C. The resulting supernatants were utilized for protein quantification using the Bradford assay (Bio-Rad Laboratories, Hercules, CA, USA). For each sample, 20 µL containing 15 µg of protein was subjected to sodium dodecyl sulfate-polyacrylamide gel electrophoresis (SDS-PAGE) and transferred onto polyvinylidene difluoride (PVDF) membranes (Millipore Corp., Bedford, MA, USA). Membranes were blocked in 5% skim milk prepared with Tris-buffered saline (TBS) containing 0.1% Tween-20 for 1 h at 23 °C before incubation overnight at 4 °C with specified primary antibodies. Following washing, membranes were exposed to secondary antibodies for 1 h at 23 °C. Quantification of protein band intensities was conducted using a LuminoGraph (Atto, Tokyo, Japan). Equal protein loading was verified using β-actin as an internal loading control. Adsorption controls and primary antibody omission controls were not included in the present study. Details concerning the antibodies employed in this study are summarized in Table 1.

### 2.8. Statistical Analysis

Data are expressed as mean ± standard deviation (SD). The results of this study were analyzed with consideration of data distribution characteristics. Given the limited sample size, non-parametric statistical methods that do not rely on assumptions of normality were used as the primary analytical approach, while parametric analyses were applied as supportive analyses to assess consistency of the results. Group comparisons were performed using the Kruskal–Wallis H-test, followed by Dunn’s multiple comparison test when the overall test was significant. For reference, parametric one-way analysis of variance (ANOVA) followed by Dunnett’s multiple comparison test was also applied, and the direction and statistical significance of the results were consistent between the two approaches. Statistical analyses were performed using Prism 7.04 (GraphPad Software Inc., San Diego, CA, USA), and a *p* value < 0.05 was considered statistically significant.

## 3. Results

### 3.1. High-Performance Liquid Chromatography (HPLC) Analysis

**Figure 1:**
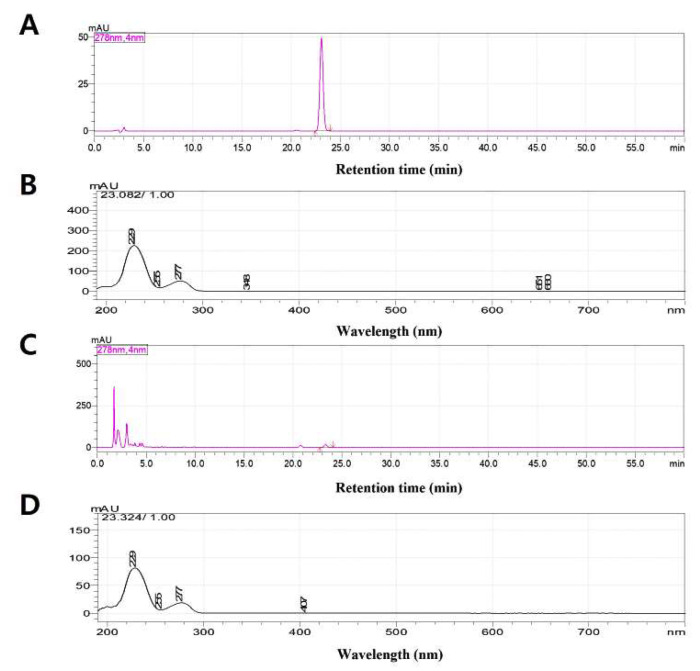
HPLC chromatograms and PDA spectra of magnolin standard and MKE. (**A**,**B**) Chromatogram and PDA spectrum of the magnolin standard. (**C**) Chromatogram of MKE. (**D**) PDA spectrum of the major peak observed in MKE.

Figure 1 presents a representative HPLC chromatogram of MKE. The chromatographic peak corresponding to magnolin was distinctly resolved from other components within the extract, verifying its appropriateness as a reference marker. Consequently, magnolin was selected as a chemical indicator for quality assessment of MKE. The retention time and PDA spectral pattern of this peak were consistent with those obtained from the magnolin standard.

### 3.2. Results of Micro-CT Analysis

**Figure 2:**
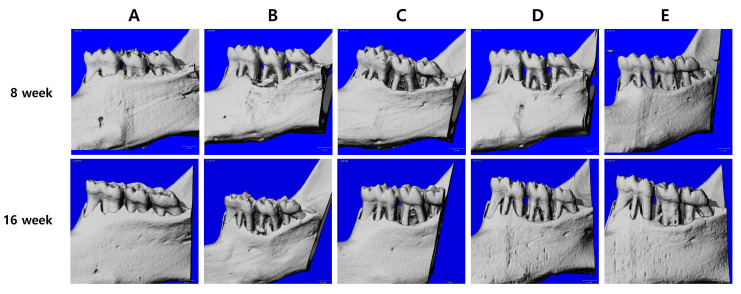
Effect of MKE on alveolar bone loss in ligature-induced periodontitis rats. Three-dimensional micro-computed tomography was used to analyze bone surrounding the three molars, and representative images from each experimental group at weeks 8 and 16 are shown. (**A**) Non-ligature control. (**B**) Ligature control. (**C**) Ligature + doxycycline 20 mg/kg. (**D**) Ligature + MKE 100 mg/kg. (**E**) Ligature + MKE 400 mg/kg. The scale bar = 1.0 mm.

After eight weeks of MKE or doxycycline administration in rats with ligature-induced periodontitis, micro-CT analysis was performed to evaluate the cementoenamel junction to alveolar bone crest (CEJ–ABC) distance and furcation involvement at baseline (Week 8, prior to treatment) and after treatment (Week 16). Quantitative results for all experimental groups are summarized in Table 2, and representative micro-CT images obtained at Weeks 8 and 16 are shown in Figure 2.

At Week 8, no significant differences in CEJ–ABC distance or furcation involvement were observed between the non-ligature control and MKE-treated groups.

At Week 16, the ligature control group exhibited a significant increase in CEJ–ABC distance (0.098 ± 0.025 mm, *p* < 0.01) and furcation involvement (0.041 ± 0.011 mm, *p* < 0.05) compared with Week 8. In addition, both parameters were significantly greater than those of the non-ligature control group at Week 16. In contrast, the non-ligature control group showed minimal changes over time, with a slight decrease in CEJ–ABC distance (−0.007 ± 0.026 mm) and a small increase in furcation involvement (0.009 ± 0.007 mm) between Weeks 8 and 16.

The doxycycline-treated group showed significant improvements in periodontal bone parameters at Week 16 compared with Week 8. CEJ–ABC distance was significantly reduced by 0.066 ± 0.018 mm (*p* < 0.01), and furcation involvement decreased by 0.030 ± 0.007 mm (*p* < 0.001).

Similarly, MKE administration resulted in reductions in CEJ–ABC distance and furcation involvement. The MKE 100 mg/kg group showed a reduction in CEJ–ABC distance of 0.049 ± 0.055 mm (*p* < 0.05), while the decrease in furcation involvement (0.014 ± 0.003 mm) did not reach statistical significance. The MKE 400 mg/kg group exhibited more pronounced effects, with significant reductions in CEJ–ABC distance (0.061 ± 0.033 mm, *p* < 0.01) and furcation involvement (0.020 ± 0.002 mm, *p* < 0.01).

### 3.3. Evaluation of MKE on Gingival Index and Tooth Mobility in Rats with Periodontitis

**Figure 3:**
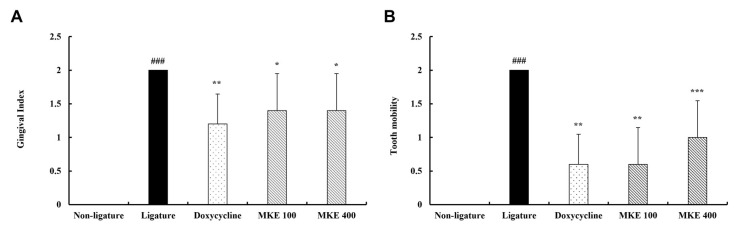
Effect of MKE on gingival index and tooth mobility. (**A**) Gingival index score measurement. (**B**) Tooth mobility score measurement. Data were collected at 16 weeks and are shown as mean ± standard deviation (*n* = 5/group). * *p* < 0.05, ** *p* < 0.01, *** *p* < 0.001 vs. ligature control group; ^###^ *p* < 0.001 vs. non-ligature control group. Non-ligature = non-ligature control group, Ligature = ligature control group, Doxycycline = ligature + doxycycline 20 mg/kg group, MKE 100 = ligature + MKE 100 mg/kg group, MKE 400 = ligature + MKE 400 mg/kg group.

At week 16, assessments of gingival index and tooth mobility were performed in rats, as shown in Figure 3. The ligature control group demonstrated mean gingival index and tooth mobility values of 2 each, which were significantly higher (*p* < 0.001) than those of the non-ligature control group. The doxycycline-treated group experienced significant reductions in gingival index and tooth mobility, with values of 1.2 and 0.6, respectively (both *p* < 0.01), compared to the ligature control group.

In the MKE 100 mg/kg group, the gingival index and tooth mobility were also significantly reduced to 1.4 (*p* < 0.05) and 0.6 (*p* < 0.01), respectively, versus the ligature control group. Likewise, the MKE 400 mg/kg group experienced significant declines in gingival index and tooth mobility, reaching 1.4 (*p* < 0.05) and 1.0 (*p* < 0.001), respectively, relative to the ligature control group.

### 3.4. Serum Analysis

**Figure 4:**
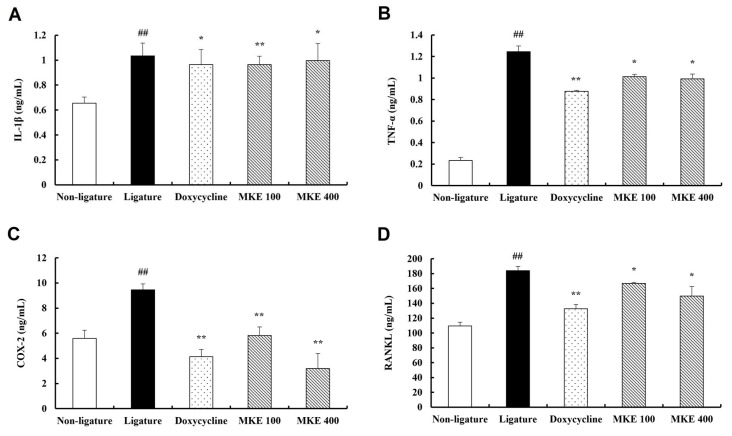
Inhibitory effects of MKE on serum inflammatory cytokines and RANKL. The concentrations of IL-1β, TNF-α, COX-2, and RANKL in serum collected at week 16 were determined. (**A**) Assessment of IL-1β levels. (**B**) Assessment of TNF-α levels. (**C**) Assessment of COX-2 levels. (**D**) Assessment of RANKL levels. Data are shown as mean ± standard deviation (*n* = 5/group). * *p* < 0.05, ** *p* < 0.01 vs. ligature control group; ^##^ *p* < 0.01 vs. non-ligature control group. Non-ligature = non-ligature control group, Ligature = ligature control group, Doxycycline = ligature + doxycycline 20 mg/kg group, MKE 100 = ligature + MKE 100 mg/kg group, MKE 400 = ligature + MKE 400 mg/kg group.

Figure 4 presents the serum analysis outcomes for rats with periodontitis. The serum concentrations of all markers were significantly increased (*p* < 0.01) in the ligature group (IL-1β: 1.03 ± 0.10 ng/mL, TNF-α: 1.25 ± 0.05 ng/mL, COX-2: 9.47 ± 0.47 ng/mL, RANKL: 183.88 ± 5.69 ng/mL) in comparison to the non-ligature control group (IL-1β: 0.66 ± 0.05, TNF-α: 0.23 ± 0.03, COX-2: 5.58 ± 0.65, RANKL: 109.58 ± 4.96). Doxycycline treatment led to a significant reduction in IL-1β serum concentrations (*p <* 0.01), and a comparable significant decrease was seen in the 100 mg/kg (0.96 ± 0.07 ng/mL, *p* < 0.01) and 400 mg/kg (1.00 ± 0.14 ng/mL, *p* < 0.05) MKE-treated groups relative to the ligature control group. Furthermore, TNF-α concentrations were significantly reduced in the 100 mg/kg (1.01 ± 0.02 ng/mL, *p* < 0.05) and 400 mg/kg (0.99 ± 0.04 ng/mL, *p* < 0.05) MKE groups compared to the ligature control group. The elevated COX-2 serum concentrations induced by ligation were markedly decreased after doxycycline administration (*p* < 0.01), and a similar significant reduction occurred in the 100 mg/kg (5.83 ± 0.68 ng/mL, *p* < 0.01) and 400 mg/kg (3.19 ± 1.20 ng/mL, *p* < 0.05) MKE-treated groups. Similarly, serum RANKL concentrations were lowered by doxycycline treatment (*p* < 0.01), and significant reductions were also apparent in the 100 mg/kg (166.87 ± 1.36 ng/mL, *p* < 0.05) and 400 mg/kg (149.70 ± 12.74 ng/mL, *p* < 0.05) MKE-treated groups compared to the ligature control group.

### 3.5. MKE Suppresses TLR4-NFκB Signaling Pathways

**Figure 5:**
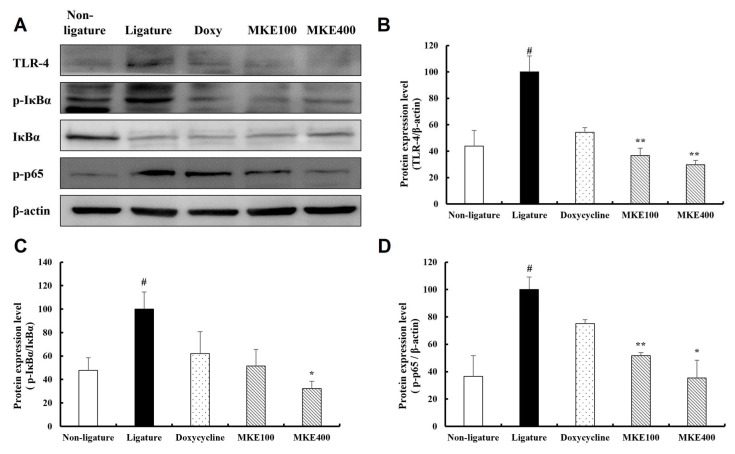
Inhibitory effect of MKE on the TLR4-NFκB signaling pathway. The expression levels of TLR-4, p-IκBα/IκBα, and p-p65/p65 in tissues collected at week 16 were evaluated. (**A**) Representative Western blot image. (**B**) Quantitative analysis of TLR-4 expression. (**C**) Quantitative analysis of the p-IκBα/IκBα expression ratio. (**D**) Quantitative analysis of the p-p65/p65 ratio. The data are expressed as mean ± standard deviation (*n* = 3/group). * *p* < 0.05, ** *p* < 0.01 compared to the ligature control group; ^#^ *p* < 0.05 versus the non-ligature control group. Non-ligature = non-ligature control group, Ligature = ligature control group, Doxycycline = ligature + doxycycline 20 mg/kg group, MKE 100 = ligature + MKE 100 mg/kg group, MKE 400 = ligature + MKE 400 mg/kg group.

Figure 5 displays the analysis of MKE’s effects on the toll-like receptor 4 (TLR-4) and NF-κB signaling pathways in periodontal tissues. The expression of TLR-4 was significantly elevated (*p* < 0.05) by 127.66 (±20.94)% in the periodontitis-induced control group relative to the normal group. In contrast, administration of MKE at 100 mg/kg and 400 mg/kg produced a marked reduction in TLR-4 expression by 63.21 (±9.42)% and 70.25 (±5.74)%, respectively (*p* < 0.01), relative to the ligature control group, showing comparable reductions to those achieved in the doxycycline-treated group (45.63 ± 5.97% reduction, *p* < 0.05).

For the NF-κB pathway, the levels of phosphorylated inhibitor of κBα (p-IκBα)/IκBα and phosphorylated p65 (p-p65)/p65 were both significantly increased (*p* < 0.05) by 109.19 (±25.19)% and 173.00 (±15.89)%, respectively, in the periodontitis-induced control group compared to the normal group. However, the MKE-treated groups showed a significant reduction in these protein levels relative to the ligature control group. Notably, the p-IκBα/IκBα ratio was significantly diminished by 67.72 (±10.76)% (*p* < 0.05) in the 400 mg/kg MKE group, while p-p65 expression fell by 48.33 (±3.84)% (*p* < 0.01) and 64.62 (±22.53)% (*p* < 0.05) in the 100 mg/kg and 400 mg/kg MKE groups, respectively.

### 3.6. MKE Suppresses the Expression of Inflammation-Associated Proteins

**Figure 6:**
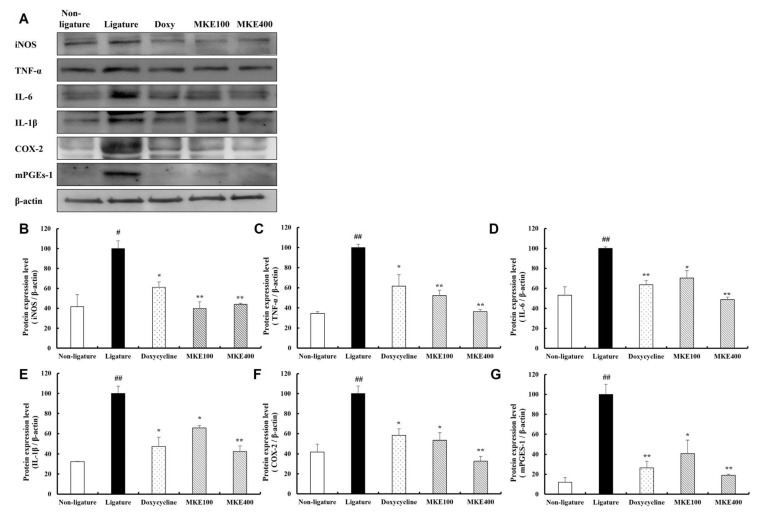
Inhibitory effect of MKE on inflammation-related markers. The expression levels of iNOS, TNF-α, IL-6, IL-1β, COX-2, and mPGES-1 in tissues collected at week 16 were determined. (**A**) Representative Western blot image. (**B**) Quantitative evaluation of iNOS expression. (**C**) Quantification of TNF-α expression. (**D**) Measurement of IL-6 expression. (**E**) Quantification of IL-1β expression. (**F**) Analysis of COX-2 expression levels. (**G**) Measurement of mPGES-1 expression. Results are shown as mean ± standard deviation (*n* = 3/group). * *p* < 0.05, ** *p* < 0.01 compared to the ligature control group; ^#^ *p* < 0.05, ^##^ *p* < 0.01 compared to the non-ligature control group. Non-ligature = non-ligature control group, Ligature = ligature control group, Doxycycline = ligature + doxycycline 20 mg/kg group, MKE 100 = ligature + MKE 100 mg/kg group, MKE 400 = ligature + MKE 400 mg/kg group.

Figure 6 presents the analysis of how MKE influences the expression of inflammation-associated proteins in periodontal tissues. The levels of key inflammation-related proteins, including inducible nitric oxide synthase (iNOS), TNF-α, IL-6, IL-1β, COX-2, and microsomal prostaglandin E synthase-1 (mPGES-1), were found to be markedly increased in the periodontitis-induced control group by 140.12 (±13.44)%, 189.69 (±5.71)%, 88.29 (±2.79)%, 209.89 (±12.58)%, and 726.28 (±17.68)%, respectively (*p* < 0.01), in comparison to the normal group. Conversely, administration of MKE at 100 mg/kg and 400 mg/kg significantly attenuated the expression of these proteins relative to the ligature control group, demonstrating reductions similar to those achieved with doxycycline treatment. More specifically, iNOS expression was reduced by 60.15 (±11.58)% at 100 mg/kg and 55.95 (±1.63)% at 400 mg/kg (both *p* < 0.01), while TNF-α levels decreased by 47.75 (±9.24)% at 100 mg/kg and 63.73 (±3.48)% at 400 mg/kg (both *p* < 0.01). In addition, IL-6 expression was diminished by 29.64 (±12.70)% at 100 mg/kg (*p* < 0.05) and 51.17 (±4.18)% at 400 mg/kg (*p* < 0.01), and IL-1β levels were decreased by 34.28 (±4.27)% at 100 mg/kg (*p* < 0.05) and 57.62 (±9.88)% at 400 mg/kg (*p* < 0.01). Furthermore, COX-2 expression was lowered by 46.62 (±13.24)% at 100 mg/kg (*p* < 0.05) and by 67.34 (±8.28)% at 400 mg/kg (*p* < 0.01), while mPGES-1 showed the greatest reduction, declining by 59.15 (±22.96)% at 100 mg/kg (*p* < 0.05) and 81.12 (±2.24)% at 400 mg/kg (*p* < 0.01).

### 3.7. MKE Suppresses MMP Expression

**Figure 7:**
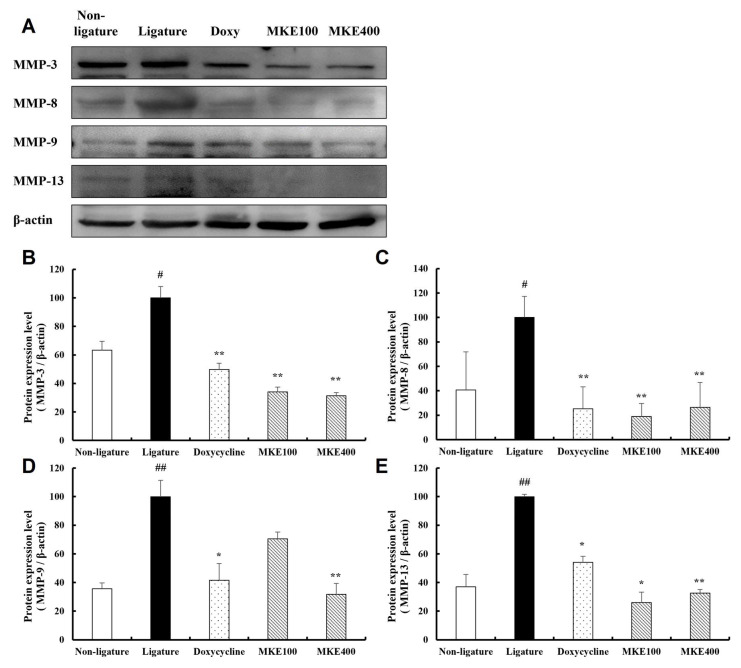
Inhibitory effect of MKE on matrix metalloproteinases. The levels of MMP-3, MMP-8, MMP-9, and MMP-13 expression in tissues collected at week 16 were assessed. (**A**) Representative image from western blot analyses. (**B**) Quantification of MMP-3 expression. (**C**) Quantification of MMP-8 expression. (**D**) Quantification of MMP-9 expression. (**E**) Quantification of MMP-13 expression. Data are shown as mean ± standard deviation (*n* = 3/group). * *p* < 0.05, ** *p* < 0.01 vs. ligature control group; ^#^ *p* < 0.05, ^##^ *p* < 0.01 vs. non-ligature control group. Non-ligature = non-ligature control group, Ligature = ligature control group, Doxycycline = ligature + doxycycline 20 mg/kg group, MKE 100 = ligature + MKE 100 mg/kg group, MKE 400 = ligature + MKE 400 mg/kg group.

Figure 7 illustrates the impact of MKE on the expression of MMPs, which are involved in the degradation of collagen and extracellular matrix, within periodontal tissues. MMP levels were significantly elevated in the periodontitis-induced control group when compared to the normal group. In contrast, treatment with MKE at both 100 mg/kg and 400 mg/kg resulted in significant downregulation of MMP expression compared to the ligature control, showing similar effects as the doxycycline group. In the group receiving 100 mg/kg MKE, MMP-3 expression decreased by 65.88 (±5.86)% (*p* < 0.01), MMP-8 decreased by 81.06 (±10.78)% (*p* < 0.01), MMP-9 decreased by 29.58 (±8.41)% (no significance), and MMP-13 decreased by 74.06 (±26.84)% (*p* < 0.05). Likewise, in the 400 mg/kg MKE group, MMP-3 expression was reduced by 68.63 (±3.66)%, MMP-8 by 73.53 (±20.36)%, MMP-9 by 68.28 (±13.12)%, and MMP-13 by 67.33 (±18.89)% (all *p* < 0.01).

### 3.8. MKE Promotes the Expression of Collagen Proteins

**Figure 8:**
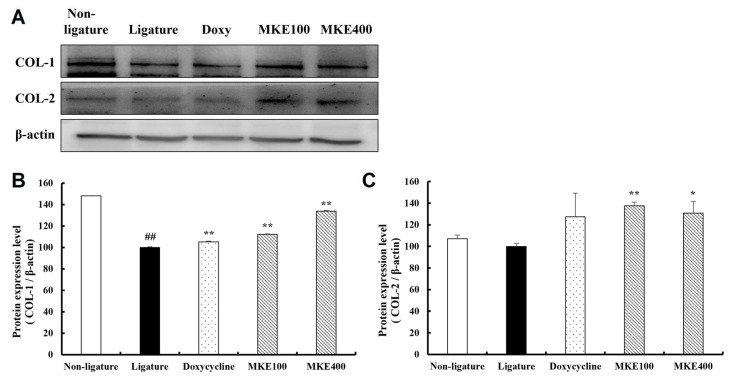
Enhancing effect of MKE on collagen proteins. The expression levels of COL-1 and COL-2 in tissues collected at week 16 were determined. (**A**) Representative results from Western blotting. (**B**) Quantitative analysis of COL-1 expression. (**C**) Quantitative analysis of COL-2 expression. Data are expressed as mean ± standard deviation (*n* = 3/group). * *p* < 0.05, ** *p* < 0.01 vs. ligature control group; ^##^ *p* < 0.01 vs. non-ligature control group. Non-ligature = non-ligature control group, Ligature = ligature control group, Doxycycline = ligature + doxycycline 20 mg/kg group, MKE 100 = ligature + MKE 100 mg/kg group, MKE 400 = ligature + MKE 400 mg/kg group.

Figure 8 presents the analysis results regarding the effects of MKE on collagen protein expression in periodontal tissues. In the periodontitis-induced control group, the expression level of collagen type I (COL-1) protein was significantly reduced by 32.51% (±0.20) compared to the normal group. Although collagen type II (COL-2) protein expression decreased by 6.63% (±4.66) compared to the normal group, this decrease was not statistically significant. In contrast, treatment with MKE (100 mg/kg and 400 mg/kg) led to a significant increase in collagen expression compared to the ligature control group, with outcomes comparable to those observed in the doxycycline-treated group. Specifically, in the 100 mg/kg MKE group, COL-1 and COL-2 expression levels increased significantly by 12.12 (±1.13)% and 32.57 (±5.82)%, respectively (both *p* < 0.01). Likewise, in the 400 mg/kg group, COL-1 and COL-2 expression levels were significantly elevated by 33.76 (±1.13)% (*p* < 0.01) and 30.87 (±18.36)% (*p* < 0.05), respectively.

## 4. Discussion

The findings of this study indicate that MKE confers substantial protective effects against tissue damage and alveolar bone resorption caused by periodontitis.

Specifically, MKE administration reduced inflammatory cytokine expression, suppressed NF-κB pathway activation, downregulated MMPs, and enhanced collagen-related protein expression, as demonstrated by the present experimental results.

Chronic periodontitis is marked by persistent inflammation, resulting in ongoing destruction of periodontal tissues and alveolar bone loss [2,4]. In the present study, MKE significantly decreased the levels of key inflammatory mediators such as iNOS, TNF-α, IL-6, IL-1β, COX-2, and mPGES-1 in the periodontitis-induced rat model. These findings are consistent with previous reports describing the anti-inflammatory activities of Magnolia species [21,22,23].

The marked suppression of pro-inflammatory mediators in periodontal tissue (TNF-α, IL-1β, IL-6, COX-2, iNOS) was accompanied by reductions in serum concentrations of IL-1β, TNF-α, and COX-2. This concordance suggests that amelioration of local periodontal inflammation reduces systemic biomarker levels; however, several important limitations must be acknowledged. First, serum measurement of only four cytokines provides a limited assessment of systemic inflammation, which involves hundreds of mediators and multiple organ systems [24]. Second, previous studies in periodontitis have demonstrated weak correlations between serum and gingival crevicular fluid (GCF) cytokine levels, suggesting that serum biomarkers may not accurately reflect local periodontal inflammation [25]. Third, the present study lacked microbiological assessment; therefore, serum marker reductions could result from reduced bacterial burden and associated LPS translocation rather than direct anti-inflammatory activity [26]. Without GCF analysis, comprehensive serum profiling, and microbiological data, claims about MKE’s systemic anti-inflammatory effects remain speculative. The serum marker reductions are more appropriately interpreted as accompanying improvements in local periodontal disease control rather than evidence of direct systemic anti-inflammatory activity.

Micro-CT analysis demonstrated that MKE significantly reduced alveolar bone loss and furcation involvement in periodontitis-induced rats. The CEJ–ABC distance, an important measure of alveolar bone resorption, increased significantly in the ligature control group, reflecting ongoing bone deterioration. In contrast, MKE administration resulted in a dose-dependent decrease in CEJ–ABC distance, indicating its capacity to preserve alveolar bone architecture. Furthermore, tooth mobility, a primary indicator of periodontal stability, was significantly lower in the MKE-treated groups, thereby supporting its effectiveness in maintaining periodontal health.

Serum analysis showed that RANKL concentrations were notably lower in MKE-treated rats relative to the ligature control group. RANKL functions as a major regulator of osteoclastogenesis, promoting excessive bone resorption during periodontitis [27,28]. The reduction in RANKL levels suggests that MKE may attenuate osteoclast-mediated alveolar bone resorption in this model. Collectively, these data support the hypothesis that MKE retards periodontitis progression by modulating both inflammatory and bone resorption mechanisms.

Among the primary findings, the suppression of the NF-κB signaling pathway by MKE was especially noteworthy. NF-κB serves a pivotal role in periodontitis progression by controlling the expression of pro-inflammatory cytokines and mediators involved in immune responses [29]. The present study demonstrated that MKE significantly reduced the phosphorylation of IκBα and p65, which are critical markers of NF-κB activation. These findings indicate that MKE attenuates periodontal inflammation, at least in part, through the inhibition of NF-κB activation. This mechanism aligns with earlier studies reporting that NF-κB inhibition results in decreased periodontal inflammation and enhances tissue integrity [30].

In addition to these findings, the presence of well-characterized bioactive lignans in *Magnolia kobus* extract provides further mechanistic support for its observed anti-inflammatory effects. Among these compounds, magnolin has been reported to inhibit LPS-induced activation of the TLR4/NF-κB and MAPK signaling pathways in RAW 264.7 macrophages, leading to significant reductions in TNF-α, IL-1β, and IL-6 expression [17]. Similarly, magnolol, another lignan derivative from Magnolia species, suppresses pro-inflammatory mediator production through blockade of NF-κB and MAPK phosphorylation [31]. Neolignans isolated from *Magnolia obovata* fruits were also shown to decrease nitric oxide (NO) generation in LPS-stimulated macrophages without cytotoxicity [32]. Furthermore, Epimagnolin A derived from *Magnolia fargesii* inhibited IL-6 production by modulating the p38/NF-κB and AP-1 pathways in human THP-1 cells [33]. These previous studies collectively indicate that magnolin and related lignan or neolignan constituents in MKE likely play a key role in suppressing inflammatory signaling cascades, thereby contributing to the protective effects observed against periodontitis in the present study. However, only magnolin was quantified in MKE (1.5%), and the contents and bioavailability of other lignans were not determined in the present study; therefore, any attribution of the in vivo effects of MKE to specific lignan or neolignan constituents should be regarded as speculative and hypothesis-generating rather than definitive.

MMPs are essential mediators of collagen and extracellular matrix degradation, which contributes to periodontal tissue destruction [34,35]. In this investigation, MKE markedly suppressed the expression of MMP-3, MMP-8, MMP-9, and MMP-13, an effect that corresponds to reduced collagen breakdown and enhanced maintenance of periodontal tissue structure. Additionally, MKE treatment resulted in elevated levels of COL-1 and COL-2 proteins, reinforcing its function in periodontal tissue protection and repair. These findings indicate that MKE contributes to the preservation of periodontal tissue structure by limiting matrix degradation.

While the present findings support a role for MKE in modulating inflammatory and bone-resorptive pathways, including NF-κB signaling and MMP expression, it should be noted that the ligature-induced periodontitis model involves complex interactions between host inflammatory responses and bacterial accumulation. Therefore, in addition to the direct molecular effects observed, secondary factors such as reduced plaque-associated bacterial burden, altered host–microbe interactions, or non-specific systemic influences related to MKE administration (e.g., modulation of stress responses or nutritional and metabolic effects) may have contributed to the overall attenuation of periodontal tissue destruction.

In addition, a limitation of this study is the absence of histological analysis. While micro-CT and molecular data provided quantitative and mechanistic insights into alveolar bone loss and inflammatory responses, cellular inflammatory infiltration, osteoclast presence, and tissue-level changes could not be directly confirmed. Future studies incorporating histological evaluation will be required to further validate these findings.

Taken together, these results indicate that MKE exerts anti-inflammatory and bone-protective effects in experimental periodontitis.

## 5. Conclusions

**Figure 9:**
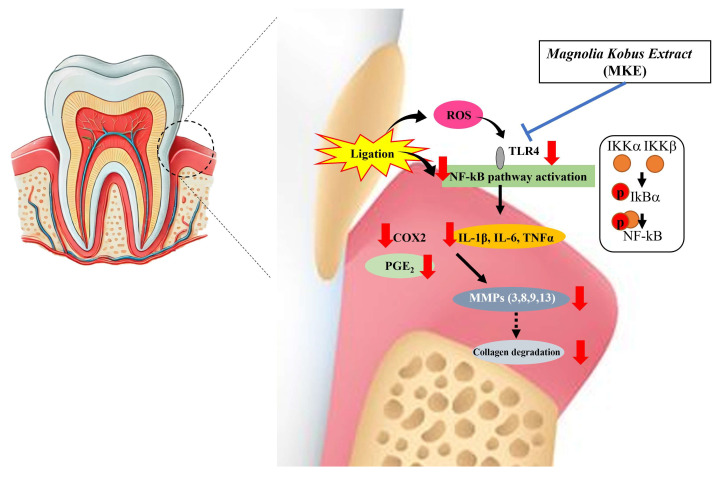
The postulated mechanism underlying the anti-periodontitis activity of MKE. MKE = *Magnolia kobus* DC. extract, ROS = reactive oxygen species, TLR-4 = toll-like receptor 4, p-IκBα = phosphorylated inhibitor of κBα, IκBα = inhibitor of κBα, IL-1β = interleukin-1β, IL-6 = interleukin-6, TNF-α = tumor necrosis factor-α, COX-2 = cyclooxygenase-2, MMPs = matrix metalloproteinases.

This study provides evidence that MKE substantially reduces inflammation, curtails alveolar bone loss, and maintains periodontal tissue architecture in a ligature-induced periodontitis model. These effects were associated with the inhibition of NF-κB signaling, reduced inflammatory cytokine production, and suppression of MMP expression. Micro-CT assessment further demonstrated that MKE effectively decreased CEJ–ABC distance and improved furcation involvement, and reductions in tooth mobility indicate a supportive effect on periodontal stability. Collectively, these results demonstrate that MKE limits gingival tissue injury and alveolar bone destruction in experimental periodontitis (Figure 9).

## Figures and Tables

**Table 1:** Information of specific antibodies used in this study.

Antibodies	Distributor
Toll-like receptor 4 (TLR-4)	Cell Signaling Technology (Danvers, MA, USA)
Inhibitor of κBα (IκBα)	Cell Signaling Technology (Danvers, MA, USA)
Phosphorylated inhibitor of κBα (p-IκBα)	Cell Signaling Technology (Danvers, MA, USA)
Phosphorylated p65 (p-p65)	Cell Signaling Technology (Danvers, MA, USA)
Inducible nitric oxide synthase (iNOS)	Cell Signaling Technology (Danvers, MA, USA)
Tumor necrosis factor-α (TNF-α)	Abcam (Cambridge, MA, USA)
Interleukin-6 (IL-6)	Abcam (Cambridge, MA, USA)
Interleukin-1β (IL-1β)	Abcam (Cambridge, MA, USA)
Cyclooxygenase-2 (COX-2)	Cell Signaling Technology (MA, USA)
Microsomal prostaglandin E synthase-1 (mPGEs-1)	Abcam (Cambridge, MA, USA)
Matrix metalloproteinase-3 (MMP-3)	Abcam (Cambridge, MA, USA)
Matrix metalloproteinase-8 (MMP-8)	Abcam (Cambridge, MA, USA)
Matrix metalloproteinase-9 (MMP-9)	Abcam (Cambridge, MA, USA)
Matrix metalloproteinase-13 (MMP-13)	Abcam (Cambridge, MA, USA)
Collagen type I (COL-1)	Cell Signaling Technology (Danvers, MA, USA)
Collagen type II (COL-2)	Santa Cruz Biotechnology (Santa Cruz, CA, USA)
β-actin	Cell Signaling Technology (Danvers, MA, USA)
Horseradish peroxidase (HRP)–conjugated anti-rabbit IgG	GenDEPOT (Barker, TX, USA)

**Table 2:** Effects of MKE on the cement-enamel junction (CEJ)–alveolar bone crest (ABC) distance and furcation involvement, as assessed by micro-computed image analysis in rats with periodontitis.

Treatment	8 Weeks		16 Weeks		Week 16–Week 8	
	CEJ–ABC Distance (mm)	Furcation Involvement (mm)	CEJ–ABC Distance (mm)	Furcation Involvement (mm)	CEJ–ABC Distance (mm)	Furcation Involvement (mm)
Non-ligature control	0.758 ± 0.089	0.166 ± 0.019	0.751 ± 0.099	0.175 ± 0.024	−0.007 ± 0.026	0.009 ± 0.007
Ligature control	1.638 ± 0.161 ^##^	0.335 ± 0.018 ^##^	1.736 ± 0.157 ^##^	0.377 ± 0.014 ^#^	0.098 ± 0.025 ^##^	0.041 ± 0.011 ^#^
Doxycycline	1.483 ± 0.056 *	0.348 ± 0.026	1.417 ± 0.046 **	0.318 ± 0.030 *	−0.066 ± 0.018 **	−0.030 ± 0.007 ***
MKE 100	1.587 ±0.121	0.341 ± 0.021	1.537 ± 0.107	0.326 ± 0.019 *	−0.049 ± 0.055 *	−0.014 ± 0.003
MKE 400	1.601 ± 0.082	0.328 ± 0.011	1.555 ± 0.078	0.308 ± 0.010 ***	−0.061 ± 0.033 **	−0.020 ± 0.002 **

* *p* < 0.05, ** *p* < 0.01, *** *p* < 0.001 vs. ligature control group; ^#^ *p* < 0.05, ^##^ *p* < 0.01 vs. non-ligature control group.

## Data Availability

The original contributions presented in this study are included in the article. Further inquiries can be directed to the corresponding author.

## Abbreviations

The following abbreviations are used in this manuscript:

MKE	*Magnolia kobus* DC. extract
IL-1β	Interleukin-1 β
TNF-α	Tumor necrosis factor-α
COX-2	Cyclooxygenase-2
RANKL	Receptor activator of nuclear factor κ-Β ligand
TLR-4	Toll-like receptor 4
p-IκBα	Phosphorylated inhibitor of κBα
IκBα	Inhibitor of κBα.
iNOS	Inducible nitric oxide synthase
IL-6	Interleukin-6
mPGES-1	Microsomal prostaglandin E synthase-1
MMP	Matrix metalloproteinase
COL-1	Collagen type I
COL-2	Collagen type II

## References

[1] Zhao P., Xu A., Leung W.K. (2022). Obesity, bone loss, and periodontitis: The interlink. Biomolecules.

[2] Li Y., Ling J., Jiang Q. (2021). Inflammasomes in alveolar bone loss. Front. Immunol..

[3] Cui Z., Wang P., Gao W. (2025). Microbial dysbiosis in periodontitis and peri-implantitis: Pathogenesis, immune responses, and therapeutic. Front. Cell. Infect. Microbiol..

[4] Usui M., Onizuka S., Sato T., Kokabu S., Ariyoshi W., Nakashima K. (2021). Mechanism of alveolar bone destruction in periodontitis—Periodontal bacteria and inflammation. Jpn. Dent. Sci. Rev..

[5] Araújo A.A., Pereira A.S.B.F., Medeiros C.A.C.X., Brito G.A.C., Leitão R.F.C., Araújo L.S., Guedes P.M.M., Hiyari S., Pirih F.Q., Araújo Júnior R.F. (2017). Effects of metformin on inflammation, oxidative stress, and bone loss in a rat model of periodontitis. PLoS ONE.

[6] Kornman K.S., Page R.C., Tonetti M.S. (1997). The host response to the microbial challenge in periodontitis: Assembling the players. Periodontol. 2000.

[7] Di Stefano M., Polizzi A., Santonocito S., Romano A., Lombardi T., Isola G. (2022). Impact of oral microbiome in periodontal health and periodontitis: A critical review on prevention and treatment. Int. J. Mol. Sci..

[8] Preshaw P.M., Taylor J.J. (2011). How has research into cytokine interactions and their role in driving immune responses impacted our understanding of periodontitis?. J. Clin. Periodontol..

[9] Yi X., Xiao Z., Chen J., Chen G., Ma P. (2025). Pharmacological Potential and Molecular Targets of Tetrahydrofurofuranoid Lignans from *Magnoliae flos*. Drug Des. Devel. Ther..

[10] Choi M., Kim J.K., Yoon J., Lim J., Kim K., Kim B., Park C.H., Sathasivam R., Kwon S.-J., Park S.U. (2024). Identification of metabolite changes and evaluation of biological activities in edible flowers of *Magnolia kobus* at different developmental stages. Chem. Biol. Technol. Agric..

[11] Kim Y., Lee S., Choi Y.A., Chung J.M., Kim E.N., Lee B., Kim S.-Y., Jeong G.-S., Kim S.-H. (2024). *Magnolia kobus* DC leaf ethanol extract alleviated lipopolysaccharide-induced acute lung inflammation by suppressing NF-κB and Nrf2 signaling. J. Herbmed. Pharmacol..

[12] Yang D., Ma D., Song Z., Yang M., Xu Y. (2024). The composition, antioxidant and antibacterial activity of essential oils from five species of the Magnoliaceae family. Molecules.

[13] Pan J.-X., Hensens O.D., Zink D.L., Chang M.N., Hwang S.-B. (1987). Lignans with platelet activating factor antagonist activity from Magnolia biondii. Phytochemistry.

[14] Shen Y., Pang E.C., Xue C.C., Zhao Z., Lin J., Li C.G. (2008). Inhibitions of mast cell-derived histamine release by different Flos Magnoliae species in rat peritoneal mast cells. Phytomedicine.

[15] Wang F., Zhang G., Zhou Y., Gui D., Li J., Xing T., Wang N. (2014). Magnolin protects against contrast-induced nephropathy in rats via antioxidation and antiapoptosis. Oxidative Med. Cell. Longev..

[16] Xu K., Gao Y., Yang L., Liu Y., Wang C. (2021). Magnolin exhibits anti-inflammatory effects on chondrocytes via the NF-κB pathway for attenuating anterior cruciate ligament transection-induced osteoarthritis. Connect. Tissue Res..

[17] Lee H.J., Lee S.J., Lee S.K., Choi B.K., Lee D.R. (2023). *Magnolia kobus* extract inhibits periodontitis-inducing mediators in *Porphyromonas gingivalis* lipopolysaccharide-activated RAW 264.7 cells. Curr. Issues Mol. Biol..

[18] Lee H.J., Lee D.R., Choi B.K., Yang S.H. (2019). Antiperiodontitis Effects of Magnolia biondii Extract on Ligature-Induced Periodontitis in Rats. Nutrients.

[19] Lu S.H., Huang R.Y., Chou T.C. (2013). Magnolol ameliorates ligature-induced periodontitis in rats and osteoclastogenesis: In vivo and in vitro study. Evid.-Based Complement. Altern. Med..

[20] Zhang S., Niu Y., Yang Z., Zhang Y., Guo Q., Yang Y., Zhou X., Ding Y., Liu C. (2020). Biochanin A alleviates gingival inflammation and alveolar bone loss in rats with experimental periodontitis. Exp. Ther. Med..

[21] Chao L.K., Liao P.C., Ho C.L., Wang E.I., Chuang C.C., Chiu H.W., Hung L.-B., Hua K.-F. (2010). Anti-inflammatory bioactivities of honokiol through inhibition of protein kinase C, mitogen-activated protein kinase, and the NF-κB pathway to reduce LPS-induced TNFα and NO expression. J. Agric. Food Chem..

[22] Hao K.X., Hao Y.F., Zhang J., Xu X.L., Jiang J.G. (2024). Comparative anti-cancer and anti-inflammatory activities of essential oils from the bark and flower of *Magnolia officinalis* Rehd. et Wils. Foods.

[23] Oyungerel B., Lim H., Lee C.H., Choi E.H., Li G.H., Choi K.D. (2013). Anti-inflammatory effects of *Magnolia sieboldii* extract in lipopolysaccharide-stimulated RAW264.7 macrophages. Trop. J. Pharm. Res..

[24] Hajishengallis G., Chavakis T. (2021). Local and systemic mechanisms linking periodontal disease and inflammatory comorbidities. Nat. Rev. Immunol..

[25] Zekeridou A., Mombelli A., Cancela J., Courvoisier D., Giannopoulou C. (2019). Systemic inflammatory burden and local inflammation in periodontitis: What is the link between inflammatory biomarkers in serum and gingival crevicular fluid?. Clin. Oral. Investig..

[26] Arimatsu K., Yamada H., Miyazawa H., Minagawa T., Nakajima M., Ryder M.I., Gotoh K., Motooka D., Nakamura S., Iida T. (2014). Oral pathobiont induces systemic inflammation and metabolic changes associated with alteration of gut microbiota. Sci. Rep..

[27] Boyle W.J., Simonet W.S., Lacey D.L. (2003). Osteoclast differentiation and activation. Nature.

[28] Gibertoni F., Sommer M.E.L., Esquisatto M.A.M., Amaral M.E.C.D., Oliveira C.A., Andrade T.A.M., Mendonça F.A.S., Santamaria M., Felonato M. (2017). Evolution of periodontal disease: Immune response and RANK/RANKL/OPG system. Braz. Dent. J..

[29] Sun S.C. (2017). The non-canonical NF-κB pathway in immunity and inflammation. Nat. Rev. Immunol..

[30] Liu T., Zhang L., Joo D., Sun S. (2017). NF-κB signaling in inflammation. Signal Transduct. Target. Ther..

[31] Fu Y., Liu B., Zhang N., Liu Z., Liang D., Li F., Cao Y., Feng X., Zhang X., Yang Z. (2013). Magnolol Inhibits Lipopolysaccharide-Induced Inflammatory Response by Interfering with TLR4 Mediated NF-κB and MAPKs Signaling Pathways. J. Ethnopharmacol..

[32] Seo K.H., Lee D.Y., Lee D.S., Park J.H., Jeong R.H., Jung Y.J., Shrestha S., Chung I.S., Kim G.S., Kim Y.C. (2013). Neolignans from the Fruits of Magnolia obovata and Their Inhibitory Effect on NO Production in LPS-Induced RAW 264.7 Cells. Planta Med..

[33] Chun H.-W., Kim S.-J., Pham T.-H., Bak Y., Oh J., Ryu H.-W., Oh S.-R., Hong J.-T., Yoon D.-Y. (2019). Epimagnolin A Inhibits IL-6 Production by Inhibiting p38/NF-κB and AP-1 Signaling Pathways in PMA-Stimulated THP-1 Cells. Environ. Toxicol..

[34] Luchian I., Goriuc A., Sandu D., Covasa M. (2022). The role of matrix metalloproteinases (MMP-8, MMP-9, MMP-13) in periodontal and peri-implant pathological processes. Int. J. Mol. Sci..

[35] Hashim N.T., Babiker R., Rahman M.M., Mohamed R., Priya S.P., Chaitanya N.C., Islam M.S., Gobara B. (2024). Natural bioactive compounds in the management of periodontal diseases: A comprehensive review. Molecules.

